# The Relation of the Weight of the Placenta to the Weight of the Newborn Child1Transactions of the American Association of Obstetricians and Gynecologists.

**Published:** 1908-05

**Authors:** W. P Manton

**Affiliations:** Detroit


### The Relation of the Weight of the Placenta to the Weight of the Newborn Child[1]

By W. P MANTON, M.D., Detroit.

FOR many years, because of the interchange of gases (oxygen and carbonic acid) known to take place within its substance, the placenta has been referred to as the "fetal lung." Modern investigation has shown that, besides this process, the after-birth possesses peculiar functions in the elaboration of food material from the maternal blood in such form as to furnish those elements best adapted to the nourishment and upbuilding of the fetal structures.

The similarity, in this respect, between the placenta and the female mammary gland would render the term "antenatal breast" equally applicable. We find in early postnatal life effects produced according to the development of the maternal breasts and the amount and quality of the milk which they secrete. If the analogy just mentioned holds good, we might with reason look for some evidences in the development of the child at birth as to the part played by the placenta in its nourishment during intrauterine life, and we might expect to find this placental efficiency manifested in a relationship between the weight of this organ and that of the neonatus.

It is, of course, perfectly evident that the placenta, like the mammary gland, may be poorly developed and in consequence the nourishment of the child affected in this way, or certain of the placental tissues, of no value whatever in the selection of food supply, may become over-developed so that, while the after-birth is large, its capacity for the formation of nourishment remains at normal or is even diminished. Many problems of like nature would naturally suggest themselves in the consideration of this question, some of which might be answered by a comparison of weights and averages and a study of the chemical changes taking place in the placenta, while others could not be determined by these or in any other way. It would be impossible

1 Transactions of the American Association of Obstetricians and Gynecologists.

to ascertain, for instance, why a placenta, although small, elaborates a sufficient or plus quantity of food while another larger fails to supply the proper amount. We could only surmise that the chemical changes are more active or that the functioning cell surface is larger in the one than in the other.

We are quite familiar with the fact that the weight of the neonatus may be considerably modified by the amount and quality of food ingested by the mother during the nine months of utero-gestation, but, as far as I am aware, no observations have been made as to the influence of diet on the size and weight of the placenta.

By taking the average in a number of cases we can determine the normal weight of the placenta and of the newborn child at term, and thus obtain the weight-ratio between the two. We can also ascertain whether multiparity or sex exert an influence on this ratio, and altogether we can arrive at some idea as to the probable work done by the placenta by a comparison of its weight and that of the child in series of cases. In other words, we may attempt to establish the fact as to whether a large placenta means a large child or whether the size of the former has really little to do with the weight of the latter.

In order first of all to get at the average weight of both placenta and child, I have taken four hundred cases from the records of the Woman's Hospital and have used the results obtained as a standard for comparison.[1] These cases were all normal and the children, with two or three exceptions, survived for a period of at least ten days following delivery.

Taking the four hundred cases, we find that the average weight of the child is seven pounds and three ounces, while that of the placenta is one pound and three ounces—a ratio of six to one. If we now take the child pound by pound from the smallest to the largest born at term, we find that the placental weight, with one exception, gradually increases with that of the neonatus.

Thus of the whole number of cases:

| | | | | | | | | | | |
|---|---|---|---|---|---|---|---|---|---|---|
| Children | weighing | 3-4 lbs. | have | an | average | placental | weight | of | 16 | oz. |
| “ | “ | 4-5 | “ | “ | “ | “ | “ | “ | 13 13-16 | “ |
| “ | “ | 5-6 | “ | “ | “ | “ | “ | “ | 16 35-45 | “ |
| “ | “ | 6-7 | “ | “ | “ | “ | “ | “ | 17 72-94 | “ |
| “ | “ | 7-8 | “ | “ | “ | “ | “ | “ | 19 6-77 | “ |
| “ | “ | 8-9 | “ | “ | “ | “ | “ | “ | 20 42-70 | “ |
| “ | “ | 9-10 | “ | “ | “ | “ | “ | “ | 21 1-6 | “ |
| “ | “ | 10-11 | “ | “ | “ | “ | “ | “ | 25 3-8 | “ |

It is not evident why children of between 4 and 5 pounds should have a placental weight so markedly below the average.

1 The selection of four hundred absolutely normal cases from the hospital records implies a very considerable amount of work, and I am under obligations to Dr. Mary G. Haskins and House Physician, Dr. Julia A. Wood, for their careful examination of a large number of clinical charts from which these cases were taken.

Theoretically, it is possible that the diminished weight of the placenta explains the lessened weight of the child, the latter failing to grow, although healthy, on account of diminished nourishment, or that in these individual cases the normal weight of the child having been reached, some inhibitory influence is exerted on the placenta whereby its production of food supply is limited only to the actual demands of the fetus. It might be entirely possible that the placenta from its complete formation contains less gross substance but a larger and more active cell area, so that, had these children been born prematurely or, on the other hand, had they passed the 280 days limit, the placental weight would have remained the same in either instance. We often see small people who eat inordinately and yet never put on flesh; and the small and insignificant breast may secrete large and often enormous quantities of milk.

Coming now to the influence of multiparity on the weight of the placenta and child, we find that the weight of the average offspring, regardless of sex, of primiparous mothers is *seven pounds and one ounce,* and that of the placenta is *eighteen ounces and one-half*, a slight diminution in the normal average weight of both.

In the instance of the multiparous mother the converse obtains; the weight of the child is augmented 7 pounds and 4 3-8 ounces, while the placenta remains at the average weight.

Sex appears to exert some influence as regards the weight of the child, but has little effect upon that of the placenta. Thus the average weight of the male child is 7 pounds 4 ounces, that of the female 7 pounds 1 ounce. The respective placentas weight 18 2-31 ounces and 18 2-3+ ounces.

It is interesting to note that among the four hundred cases the largest child, a male, weighed 10 pounds and 8 ounces (placenta) weight 28 ounces; multiparous mother); the smallest child, a female, 3 pounds (placental weight 16 ounces; mother not stated.) The largest placenta weighed 40 ounces (male child, 8 pounds 11 ounces; primiparous mother); the smallest placenta weighed 6 ounces, (female child, 6 pounds 12 ounces; primiparous mother).

Conclusions: The figures above presented are interesting on many accounts. They at least indicate that, as a rule, the development of the placenta goes forward with that of the child, and its size may be taken ordinarily as an index to the weight development of the latter. It is further shown that, while there may be individual variations, in any given number of cases, these will not be sufficiently numerous to greatly influence the normal weight ratio between child and placenta, that is, 6+ : 1.

32 ADAMS AVE., WEST.

